# Microstructure, Microhardness and Corrosion Resistance of WE43 Alloy Based Composites after High-Pressure Torsion

**DOI:** 10.3390/ma12182980

**Published:** 2019-09-15

**Authors:** Petr Straumal, Natalia Martynenko, Askar Kilmametov, Aleksey Nekrasov, Brigitte Baretzky

**Affiliations:** 1 A.A. Baikov Institute of Metallurgy and Materials Science RAS, 119334 Moscow, Russia; straumal.peter@yandex.ru (P.S.); nataliasmartynenko@gmail.com (N.M.); 2 Institute of new materials and nanotechnologies, National University of Science and Technology «MISiS», 119049 Moscow, Russia; 3 Institute of Nanotechnology, Karlsruhe Institute of Technology, 76344 Eggenstein-Leopoldshafen, Germany; brigitte.baretzky@kit.edu; 4 Institute of Experimental Mineralogy, RAS, 142432 Chernogolovka, Russia; alexei.nekrasov@iem.ac.ru

**Keywords:** nanocrystalline metals, magnesium alloys, nanocrystalline oxides, severe plastic deformation, high-pressure torsion

## Abstract

The structure and properties of a composite consisting of Mg-Y-Nd-Zr alloy (WE43) and various oxides are studied. The particles of the WE43 powder were coated by the nanocrystalline oxide layer by means of a wet chemical deposition process. After that the powder is compressed into solid samples and deformed using high-pressure torsion at room temperature. A second phase is present, both, in pure WE43 alloy and in the one with deposited oxides. We observed that the modification of the alloy by the oxide layer deposition and deformation by high-pressure torsion changes the phase composition and properties of the samples. The samples modified by TiO_2_ showed the best microhardness and corrosion resistance.

## 1. Introduction

The properties of nanocrystalline metallic materials, such as mechanical characteristics, corrosion rate, and others, came to the fore in the last few decades. Nanocrystalline materials showed unusual functional properties because they contain an extremely high proportion of atoms located at the grain boundaries and other defects in the crystal structure. The WE43 alloy containing rare earth metals (REM) is one of the most popular medical magnesium alloys [1]. Alloying with REM improves the corrosion resistance of magnesium [2] and also increases its strength. Another method of improving the strength characteristics is severe plastic deformation (SPD), which leads to the formation of ultrafine-grained structure in magnesium and its alloys [3]. This structure provides significant hardening [3,4,5,6] and also leads to an increase in the corrosion resistance and a decrease in gas evolution.

Among all the methods of obtaining nanocrystalline alloys, high-pressure torsion (HPT), which belongs to the family of methods of severe plastic deformation occupies a special place because it demonstrates a number of advantages in comparison with its competitors, for example, it makes it possible to produce nanocrystalline materials in relatively large volumes from both pure metals and alloys.

The goal of this work is to investigate the possibility of altering the strength and corrosion properties of the WE43 alloy (a commercially available bioresorbable magnesium alloy Mg-3.56%Y-2.20%Nd-0.47%Zr, wt.% and traces of other rare-earth elements) by introducing oxide particles of various metals into the alloy and processing it by SPD. For this, the phase composition and properties of severely plastically deformed metal-ceramic composites based on the magnesium alloy WE43 modified with aluminum, titanium, magnesium and zinc oxides were studied.

Usually, the Mg-based composites can be fabricated by the casting or ball-milling process. The casting methods can be used to fabricate large parts at a low cost but the main problem is the occurrence of coarse microstructural features and comparatively large grain sizes in the final samples, resulting in low strength. Moreover, the reactions between the Mg matrix and the reinforcements cause the degradation of the composites. Another difficulty arising in the production of Mg composites by casting methods is that it is complicated to introduce fine reinforcement particles into the melt, especially when the volume fraction of the reinforcement is large. Usually, large ceramic particles with a size of a few micrometers are added. The process is more complicated when the particle size is much smaller than a tenth of a micrometer or when it is of nanometer size. Therefore, ball-billing is often chosen as the preparation method for Mg-alloys and second component composites.

Numerous Mg-oxide composites were produced by the ball-milling technique. Zhenglong et al. produced a nanocrystalline composite of Mg–3Ni–2NiO (wt.%) by mechanical milling with nickel nano-powders under the hydrogen pressure [7]. Song et al. prepared a milled melt-spun Mg-23.5 wt.%Ni with 10% nanocrystalline Nb_2_O_5_ [8]. Li et al. mechanical milled Mg–20 wt.% Ni–1 wt.% TiO_2_ under a hydrogen atmosphere which directly led to the composite MgH_2_, and Mg_2_NiH_4_ [9].

In this paper, the method of pyrolysis of organometallic compounds is used to deposit the oxide layer on the magnesium alloy powder. The idea is to crush the Mg alloy, cover the powder particles by a nanometer thick oxide layer, after that produce an Mg-ceramic composite by SPD. During the SPD process, the nanometer-thick oxide layer should break and mix with the Mg matrix, producing fine and well-distributed oxide particles. Earlier, we already successfully produced WE43 composites with Al_2_O_3_ and ZnO [10], now we are investigating the composites with a different proportion of Al_2_O_3_ and ZnO and, additionally, with TiO_2_ and MgO.

## 2. Experimental

First, the WE43 alloy was mechanically crushed into particles of 0.5–1 mm size. Second, thin films of nanocrystalline Al_2_O_3_, ZnO, MgO, and TiO_2_ were deposited onto WE43 particles using pyrolysis of organometallic compounds. This method has been developed under the supervision of Prof. A.A. Myatiev (NUST “MISiS”, Moscow, Russia) for the deposition of nanocrystalline single- and multi-component oxide films. By pyrolytic decomposition of organic precursors containing salts of metals and carbon acids, we produce nanocrystalline oxide films [11,12,13,14,15]. Carboxylates of metals formed by metal cations and anions of higher isomeric carboxylic acids containing α, α branched acid residues are used as a precursor. Carboxylates are applied to the surface of the substrate as a solution in a mixture of the same carboxylic acids, the anions of which are part of the carboxylates.

Extraction methods are used to prepare such solutions. As the extractant, a mixture of higher isomeric carboxylic acids (HICA) was used, the general formula of which:

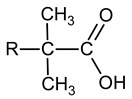


The hydrocarbon radical R can contain from one (pivalic acid) up to 12 carbon atoms.

Using this approach, the films can be deposited on various substrates. The deposited films are dense, non-porous, nanostructured, uniform, and non-textured. The chemical components in multicomponent films are distributed uniformly [16,17].

In order to deposit the oxides, the obtained powder was mixed with the organometallic solutions of aluminum or zinc and the resulting mixture was annealed in air at 400 °C. Thermal decomposition of the organic component of the solution and precipitation of films consisting of a metal oxide, the cation of which was introduced into the solution, occurs on the surface of the WE43 powder particles.

In the previous study [10], all of the later deformed WE43 powder was coated by an oxide layer. In this study, one part of the oxide-coated WE43 powder was mixed with two parts of uncoated WE43 powder. To mix these parts properly, they were mixed with heptane and put into an ultrasonic bath. After that, the mixture was completely dried on air.

After proper mixing of the coated and uncoated powders, the mixture was subjected to SPD by HPT. The powder was poured into the cavity in the lower anvil and compressed to obtain a solid pellet. Samples were deformed at a pressure of 6 GPa for 5 revolutions with 1 rpm rotation speed. The cavity in the lower anvil was of 0.2 mm depth.

The microhardness was measured using a 402 MVD Instron Wolpert Wilson Instruments tester (Aachen, Deutschland). The morphology of obtained samples and the composition of the matrix and second phase were studied by electron probe micro-analyzer (EPMA) using a Tescan Vega TS5130 MM scanning electron microscope (SEM, Brno, Czech) equipped with an Oxford Instruments energy dispersion spectrometer (Abingdon-on-Thames, UK).

The transmission electron microscopy (TEM) was performed on the FEI Titan 80-300 Transmission Electron Microscope (Thermo Fisher Scientific, Waltham, MA, USA) with a high-angle annular dark-field scanning transmission electron microscopy (HAADF-STEM) and EDAX S-UTW energy dispersive X-ray analysis (EDX) detectors.

The study of the corrosion properties was carried out using the method of measuring the volume of hydrogen released during corrosion [18]. As a test medium, a solution of 0.9% NaCl in distilled water was used (physiological solution, pH = 7). Samples were prepared manually by mechanical grinding on abrasive paper (from P800 to P2500). The tests of hydrogen evolution were carried out at room temperature and at 37 °C. The test sample was placed in a solution and covered with a funnel and a burette filled with corrosive medium. The sample remained in a corrosive environment for a day, after which the volume of hydrogen released was measured. The corrosion rate (mL/(cm^2^ * day)) for the method of hydrogen evolution was calculated by the Equation (1):

RΔv = VH/S * T(1)

where VH is the volume of released hydrogen, ml; S is the surface area of the sample, cm^2^; T—time, days.

## 3. Results and Discussion

**Figure 1:**
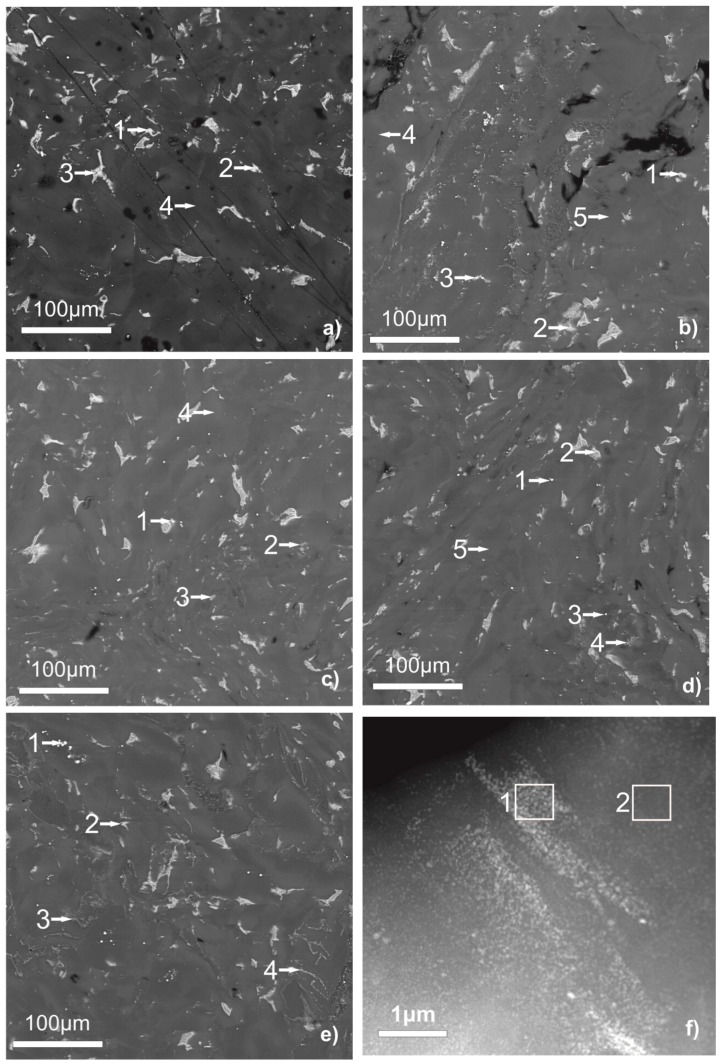
SEM micrographs of WE43 samples unmodified (**a**) [10], modified with aluminum oxide (**b**), titanium oxide (**c**), magnesium oxide (**d**), and zinc oxide (**e**) after deformation by high-pressure torsion. STEM micrograph of WE43 sample modified with aluminum oxide (**f**).

Figure 1 shows the results of a study of the obtained samples by SEM with an EPMA analysis of the matrix composition and inclusions. Table 1 shows the measured compositions of inclusions corresponding to the numbered points in Figure 1.

The sample made only from the WE43 alloy, in the previous study [10], consisted of the Mg matrix with some Y addition and low Nd content and particles of the second phase, which hold a higher Y and Nd content.

As can be seen, the composition of a part of the second phase particles in the WE43 alloy after the HPT changes with the addition of oxides. The second phase particles in the pure WE43 alloy after HPT contain only the rare earth elements from the alloy. After modifying the WE43 alloy by oxides prior to HPT, a part of the second phase particles in the samples contains Al, Ti, or Zn from the oxide; It is observed as in SEM with a lower resolution, as in HAADF-STEM with a higher resolution. The uncertainty for the Al content in the TEM investigation is 0.05 at.%. The second phase of the samples with the addition of magnesium oxide, sometimes, shows a higher oxygen, Zr, or Nd content. Besides that, the samples with the addition of zinc oxide contain thin layers of the second phase on the borders of the initial particles or grain agglomerates, thus providing a higher strength.

Under the action of SPD, the mobility of atoms and the mass transfer rate are increased to the extent typical for diffusion during annealing at elevated temperatures. This leads to the mixture of the magnesium alloy WE43 with oxides added prior to the SPD.

**Figure 2:**
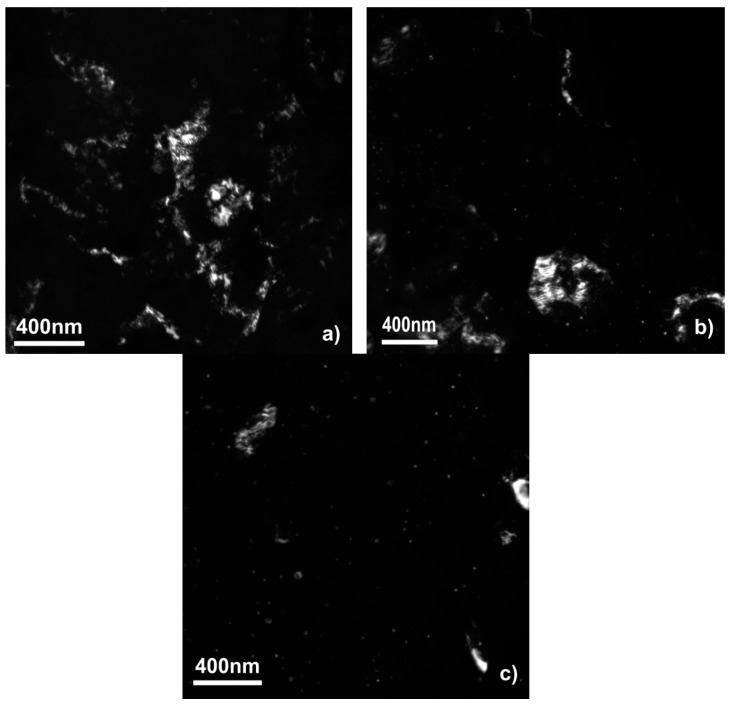
Dark-field (DF) TEM micrographs of WE43 samples unmodified (**a**), modified with aluminum oxide (**b**), and zinc oxide (**c**) after deformation by high-pressure torsion.

Figure 2 shows the fine structure of the samples obtained by a dark-field TEM study. The DF TEM micrographs show that the sample made only of the WE43 alloy consists of the matrix with some second phase particles mostly in the form of bigger (100–500 nm) particles. The situation with the samples modified by the Al and Zn oxide is different. There the second phase is presented by bigger particles and highly dispersed small (down to 5 nm) equiaxed particles. Figure 1f demonstrates the fine second phase particles, which are typical for the samples with oxides after SPD. During the SPD, the oxide layer on the surface of the powder particles breaks into fine oxide particles, which act as the segregation centers for the second phase. That promotes a more dispersive second phase segregation as we showed in our previous work [10].

The microhardness of the samples after the HPT was measured as an array of 15 × 15 points over the whole area of the samples. The measured microhardness on the middle of the sample radius after HPT is, as follows: for WE43 with aluminum oxide—969 ± 34 MPa, for WE43 with titanium oxide—1,017 ± 36 MPa, for WE43 with magnesium oxide—997 ± 36 MPa, and for WE43 with zinc oxide—986 ± 32 MPa.

The microhardness measured in the previous study [10] for pure WE43 after crushing and HPT is 1299 ± 17 MPa and for WE43 with aluminum oxide is 551 ± 14 MPa, for WE43 with zinc oxide is 867 ± 21 MPa. The value for the pure deformed WE43 corresponds well with the microhardness of as-cast WE43 deformed by HPT [3]; this shows that the crushing of the alloy prior to the HPT did not affect the microhardness of the alloy. However, it should be noted that the addition of all oxides reduces the microhardness compared to the alloy deformed without oxides. Insufficient mixing of the oxides with the alloy powder particles due to their rather large size (0.5–1 mm) may be the most likely reason for this behavior. Therefore, the structure contains oxide regions and regions with a deformed alloy, which leads to a moderate decrease in the average microhardness. This is also probably the cause of why microhardness does not increase due to the pinning of dislocations by oxide particles. The use of more dispersed particles of alloy powder can be one possible way to solve this problem.

**Figure 3:**
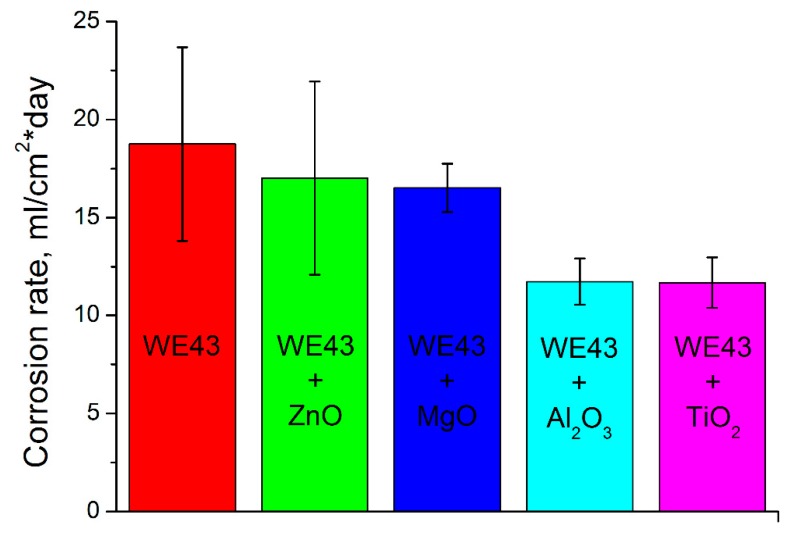
Results of pure and oxide-modified WE43 alloy corrosion tests.

The corrosion tests result in Figure 3 show us the different corrosion rates of the pure WE43 after HPT and oxide modified WE43 samples after HPT. Clearly, all oxides lower the corrosion rate but the Al_2_O_3_ and TiO_2_ perform best in that task.

## 4. Conclusions

Samples (magnesium alloy)/(oxide) composites were successfully produced. They are dense and it is possible to study their structure and properties.

The oxide metals enter the second phase particles and promote more dispersive second phase segregation. That changes the phase composition after the SPD process and, accordingly, alters the properties of the composite.

The strength of the samples increases compared to the results of the previous study; this is a result of a lower amount of modificating oxide. This reduction was planned and the results show that our study succeeded.

The corrosion resistance is higher for all modifying oxides compared to the pure WE43 after HPT but the best results were achieved for the TiO_2_. The same is true for the microhardness.

## Figures and Tables

**Table 1:** Results of the energy dispersive X-ray analysis (EDX) (conducted in SEM and TEM) of second phase precipitates in the samples of pure WE43 [10] and modified with aluminum, titanium, magnesium, and zinc oxide after high-pressure torsion (HPT) as shown in Figure 1. The composition is given in at.%.

**WE43 6GPa5N** **SEM**	**Point**	**O**	**Mg**	**Y**	**Zr**	**La**	**Nd**	**Gd**	**Dy**
	1	3.2	86.38	4.4	0.02	-	5.99	-	-
	2	3.28	87.1	4.16	0	-	5.45	-	-
	3	2.33	85.89	5.32	0.03	-	6.44	-	-
	4	6.83	91.99	0.95	0	-	0.22	-	-
**WE43 + Al_2_O_3_ 6GPa5N** **SEM**	**Point**	**O**	**Mg**	**Y**	**Al**	**La**	**Nd**	**Gd**	**Dy**
	1	2.79	84.3	4.35	0.26	0.64	7.07	0.21	0.22
	2	9.4	79.98	3.69	0.36	0.51	5.53	0.11	0.24
	3	4.69	62.15	28.07	0.1	0.27	4.06	0.8	1.67
	4	1.33	96.09	0.6	0.17	3.49	-	-	0.23
	5	1.71	97.06	0.85	0	0	0.23	0.12	0.08
**WE43 + TiO_2_ 6GPa5N** **SEM**	**Point**	**O**	**Mg**	**Y**	**Ti**	**La**	**Nd**	**Zr**	**Dy**
	1	2.48	83.85	4.85	0	0.83	7.63	0.08	0.27
	2	1.15	96.08	1.28	0.06	0.07	1.09	0.13	0.11
	3	1.53	96.73	1.12	0	0.02	0.36	0.07	0.18
	4	2.14	96.05	1.36	0	0	0.42	0.01	0.01
**WE43 + MgO 6GPa5N** **SEM**	**Point**	**O**	**Mg**	**Y**	**Zr**	**La**	**Nd**	**Gd**	**Dy**
	1	6.64	63.95	25.13	0.62	0.13	2.1	0.67	0.76
	2	2.36	86.16	4.72	0.09	0.41	5.65	0.33	0.28
	3	17.67	56.57	0	25.58	0.08	0.03	0.06	0
	4	19.49	74.3	3.3	0.15	0.09	2.33	0.12	0.23
	5	1.66	96.53	1.25	0.3	0	0.19	0	0.07
**WE43 + ZnO 6GPa5N** **SEM**	**Point**	**O**	**Mg**	**Y**	**Zn**	**Zr**	**La**	**Nd**	**Dy**
	1	15.95	16.63	0	0.04	67	0.07	0.29	0
	2	4.3	84.1	4.51	0	0.12	0.78	5.88	0.32
	3	31.67	57.15	0.29	10.71	0.01	0	0.12	0.05
	4	7.48	82.26	6.53	0.19	1.92	0.16	1.15	0.3
**WE43 + Al_2_O_3_ 6GPa5N** **STEM**	**Area**	**O**	**Mg**	**Y**	**Al**	**Zr**	**Nd**	**C**	-
	1	2.54	89.59	2.97	0.19	0.00	3.20	1.49	-
	2	1.56	97.17	0.78	0.00	0.00	0.00	0.48	-
